# Investigation of 3 dB Optical Intensity Spot Radius of Laser Beam under Scattering Underwater Channel

**DOI:** 10.3390/s20020422

**Published:** 2020-01-11

**Authors:** Wei Wang, Xiaoji Li, Sujan Rajbhandari, Yanlong Li

**Affiliations:** 1 Ministry of Education Key Laboratory of Cognitive Radio and Information Processing, Guilin University of Electronic Technology, Guilin 541004, China; baiyunchuxiu2019@outlook.com (W.W.); lylong@guet.edu.cn (Y.L.); 2 Guangxi Experiment Center of Information Science, Guilin 541004, China; 3 Institute of Future Transport and Cities, School of Computing, Electronics and Mathematics, Coventry University, Coventry CV15FB, UK; sujan.rajbhandari@coventry.ac.uk

**Keywords:** 3 dB optical intensity spot radius, scattering underwater channel, Monte Carlo simulation method

## Abstract

An important step in the design of receiver aperture and optimal spacing of the diversity scheme for an underwater laser communication system is to accurately characterize the two-dimensional (2D) spatial distribution of laser beam intensity. In this paper, the 2D optical intensity distribution and 3 dB optical intensity spot radius (OISR) are investigated due to the dominating optical intensity of laser beam being within the 3 dB OISR. By utilizing the Henyey–Greenstein function to compute the scattering angles of photons, the effects of the scattering underwater optical channel and optical system parameters on 3 dB OISR are examined based on the Monte Carlo simulation method. We have shown for the first time that in the channel with a high density of scattering particles, the divergence angle of the laser source plays a negligible role in 3 dB OISR. This is an interesting phenomenon and important for optical communication as this clearly shows that the geometric loss is no longer important for the design of receiver aperture and optimal spacing of the diversity scheme for the underwater laser communication system in the highly scattering channel.

## 1. Introduction

There had been significant interest in underwater communication for ocean exploration, environment monitoring, diver safety and other applications. Currently, acoustic, radio frequency (RF) and optical communications are considered for underwater communication. The underwater acoustic communication system suffers from limited bandwidth. Hence it is not suitable for high-speed communication. The RF spectrums suffer from extremely high attenuation in oceanic environments, limiting the communication to a very short distance. The various studies have already proven that the optical spectrum between blue and green wavelengths is one of the most suitable media to transfer information in the underwater channel due to its high bandwidth and low attenuation [1,2,3].

There are a number of studies that characterized the underwater optical channel in terms of channel attenuation, channel impulse response (CIR) and signal distribution in spatial domain using the Monte Carlo (MC) simulation method, vector radiative transfer (VRT) theory, beam spread function (BSF), radiative transfer equation (RTE), stochastic model, closed expression model, numerical model, modified Beer–Lambert (BL) law and experimental measurement. Table 1 summarizes the aforementioned methods used for characterizing the underwater optical communication and their contributions.

None of these works, however, characterize the two-dimensional (2D) spatial distribution of the laser beam on the receiving plane. The laser beams in the underwater channel experience significant random scattering. Hence, compared with the natural divergence, laser beams at the receiving plane in the underwater channel have a larger optical spot. However, the real optical receiver’s aperture tends to be significantly smaller than the whole beam spot under the scattering underwater channel. The knowledge of optical intensity distribution on the receiving plane with finite dimensions is important for designing the optical receiver aperture and optimal spacing between receivers for the spatial diversity scheme. Hence, in this paper, the optical intensity distribution of the laser beam on the receiving plane in the scattering underwater medium is studied for the first time.

In order to characterize the underwater optical channel, we defined and calculated 3 dB optical intensity spot radius (OISR) at the receiver’s plane as the dominating optical power of the laser beam be within the 3 dB OISR. The MC simulation method is often used to study the radiance transfer equation of optical waves’ propagation in the scattered media and also applied to trace the trajectory of photons. Compared with the experimental measurements, the MC approach offers flexibility to alter the optical channel and system parameters. More importantly, it can reveal the statistical characteristics of underwater optical channel accurately because an enormous number of photons are counted [9,10,12]. Hence, based on the MC simulation method, the investigation on the effects of underwater channel and optical system parameters (such as the channel type, half-aperture size of receiving plane with finite dimensions, transmission distance and divergence angle) on the 3 dB OISR is very important for the design of receiver aperture and optimal spacing of spatial diversity scheme for an underwater laser communication system. The results show that in a highly scattering channel (such as harbor seawater channel) the optical intensity distribution is no longer a Gaussian and the effects of the divergence angle on 3 dB OISR is negligible i.e., the geometric loss is no longer important for the design of receiver aperture and optimal spacing of the diversity scheme in the highly scattering underwater communication channel.

The rest of the paper is organized as follows: Section 2 describes the principle of the MC simulation method, the computational principle of 3 dB OISR is given in Section 3, simulation results and analysis are presented in Section 4 and followed by conclusions in Section 5.

## 2. Monte Carlo Simulation Method

To adopt the MC simulation method to trace the propagation trajectory of photons in seawater, six key parameters of photons are considered: (a) Coordinates $\left(x_{0},y_{0},z_{0}\right)=\left(r_{0}cos\psi_{0},r_{0}sin\psi_{0},0\right)$; (b) zenith angle ($\theta_{0}=T-\mathrm{Phai}\sqrt{-\ln(1-\xi_{1})}/2)$; (c) azimuth angle ($\psi_{0}=2\pi \xi_{2}$) for photons emission; (d) propagation distance (*d*); (e) zenith angle ($\theta_{s}$) and (f) azimuth angle ($\psi_{s}$) for each scattering event [20]. Here $r_{0}=w_{0}\sqrt{-\ln(1-\xi_{3})}$ is the distance of emission photons to the geometric center of the laser source, $w_{0}$ is the beam waist radius and T−Phai is the divergence angle of the laser beam; $\xi_{1}$, $\xi_{2}$ and $\xi_{3}$ are random number uniform on [0, 1].

### 2.1. Scattering Phase Function

Unlike the free space atmospheric environments, seawater contains massive phytoplankton, dissolved salts, mineral particles and dissolved organic matter, which induces absorption and scattering effects on the laser beam, particularly for the coastal and harbor underwater optical channel. To model the scattering of the laser beam caused by suspended particles, the volume scattering function (VSF) $\bar{\beta }\left(\theta ,\lambda \right)$ is used to characterize the scattered intensity per unit incident irradiance per unit volume of water. Assume the laser beam to be unpolarized and the seawater to be isotropic, and hence the scattering becomes angular dependent. It is presented as a fraction of scattered out intensity of the laser beam through an angle θ into a solid angle ΔΩ, and the VSF is given as [1,3]:

(1) $$\bar{\beta }\left(\theta ,\lambda \right)=\lim_{\Delta r\to 0}\lim_{\Delta \Omega \to 0}\frac{P_{s}\left(\theta ,\lambda \right)}{P_{i}\left(\theta ,\lambda \right)\Delta r\Delta \Omega }$$

where θ is the scattering angle of photons, $P_{s}\left(\theta ,\lambda \right)$ is the scattered optical power through θ into ΔΩ, $P_{i}\left(\theta ,\lambda \right)$ is the incident optical power, λ is the wavelength of the laser beam and Δr is the seawater thickness. By integrating the $\bar{\beta }\left(\theta ,\lambda \right)$ over all angles, the scattering coefficient $K_{s}\left(\lambda \right)$ is obtained as [1,3]:

(2) $$K_{s}\left(\lambda \right)=\int_{4\pi }\bar{\beta }\left(\theta ,\lambda \right)\;d\theta =2\pi \int_{0}^{\pi }\bar{\beta }\left(\theta ,\lambda \right)sin\theta \;d\theta$$

The scattering phase function (SPF) is used to describe the probability distribution of propagation direction of the scattered photons. Normalizing Equation (1) with $K_{s}\left(\lambda \right)$, the SPF is expressed as [1,3]:

(3) $$\beta \left(\theta ,\lambda \right)=\frac{\bar{\beta }\left(\theta ,\lambda \right)}{K_{s}\left(\lambda \right)}$$

There are various SPF models to represent the scattering characteristics of seawater channels, such as the Fournier–Forand function, the Henyey–Greenstein (HG) function and their modifications. Among the above-mentioned SPF functions, only the HG function can establish analytical expression between the scattering angles and the random numbers [20]. This is in favor of improving the computational accuracy and reducing the computational complexity in simulation analysis. While the HG function fails to provide very accurate results for photon scattering with small and large angles, such deviations are considered acceptable in the theoretical analysis [38]. Hence, in this paper, we adopt the HG function as SPF to compute the scattering angles, which satisfies the following equation:

(4) $$1=\int_{0}^{\pi }\beta \left(\theta ,\lambda \right)\sin\theta \;d\theta$$

where β(θ,λ) is the SPF, θ is the scattering angle of photons and λ is the wavelength of the laser beam. In this paper, a fixed wavelength λ of 532 nm is selected. Hence, β(θ,λ) can be replaced by β(θ), and the expression of the HG function is given by [39]:

(5) $$\beta_{HG}\left(\theta \right)=\frac{1-g^{2}}{4\pi {\left(1+g^{2}-2g\cos\theta \right)}^{-3/2}}$$

where *g* is the asymmetry parameter (equal to the average cosine of the scattering angle over all scattering angles).

### 2.2. Photon Propagation

#### 2.2.1. Propagation Distance

According to the definition of optical distance *L* and the Beer Law, the probability density function for the intensity attenuation of the laser beam as a function of *L* is given by [39,40,41]:

(6) $$p_{L}\left(L\right)=\exp\left(-L\right),L>0$$

Hence, the probability that a photon is absorbed and scattered between an optical distance 0 to *L* is given by [39,40,41]:

(7) $$P_{L}\left(L\right)=\int_{0}^{L}p_{L}\left(l\right)\;dl=\xi_{4}$$

where is the probability of photons travel over an optical distance of *L*, and $P_{L}\left(L\right)=\xi_{4}$ is a random number uniform on [0,1]. Consequently, $L=-\ln(1-\xi_{4})$, due to $L=K_{att}\left(\lambda \right)d$, $K_{att}\left(\lambda \right)$ is the attenuation coefficient. Hence, the photons propagation distance for each scattering event can be solved by:

(8) $$d=-\ln\left(1-\xi_{4}\right)/K_{att}\left(\lambda \right)$$

#### 2.2.2. Photon Weight

As the laser beam propagates over a distance in the seawater channel, a certain percentage of photon energy is absorbed and the rest is scattered. The energy weight of photon after scattering is given by:

(9) $$w_{post}=\mu w_{pre}$$

where $\mu =K_{s}\left(\lambda \right)/K_{att}\left(\lambda \right)$ is the albedo, $w_{pre}$ and $w_{post}$ are the pre-scattering and post-scattering energy weight, respectively. Each time the photon is scattered, the energy weight survival rate is μ. In this paper, initial energy weight is assumed to be 1. To improve computation time, $w_{post}=10^{-10}$ is set as the photon’s survival threshold, i.e., $w_{post}<10^{-10}$, the propagating photons are annihilated.

#### 2.2.3. Propagation Direction

According to $\theta_{0}$ and $\psi_{0}$ for photon emission, $\left(\mu_{x_{0}},\mu_{y_{0}},\mu_{z_{0}}\right)=\left(sin\theta_{0}cos\psi_{0},sin\theta_{0}sin\psi_{0},cos\theta_{0}\right)$ [40] is the initial direction vector. The scattering zenith angle $\theta_{s}$ and azimuth angle $\psi_{s}$ for each scattering event are given by Equation (10), and the $\theta_{s}$ is computed by the inverse of HG function given by:

(10) $$\left\{\begin{array}{c} \theta_{s}=arccos\left\{\frac{1}{2g}\left[1+g^{2}-{\left(\frac{1-g^{2}}{1+g+2g\xi_{5}}\right)}^{2}\right]\right\}\\ \psi_{s}=2\pi \xi_{6} \end{array}\right.$$

where $\xi_{5}$ and $\xi_{6}$ are random number uniform on [0,1].

Assume the unit direction vector of photons for pre-scattering is $\left(\mu_{x},\mu_{y},\mu_{z}\right)$, the direction vector of photons for post-scattering $\left(\mu_{x}^{\prime },\mu_{y}^{\prime },\mu_{z}^{\prime }\right)$ is given by [40]:

(11) $$\left[\begin{array}{c} \mu_{x}^{\prime }\\ \mu_{y}^{\prime }\\ \mu_{z}^{\prime } \end{array}\right]=\left[\begin{array}{ccc} \mu_{x}\mu_{z}/\sqrt{1-\mu_{z}^{2}} & -\mu_{y}/\sqrt{1-\mu_{z}^{2}} & \mu_{x}\\ \mu_{y}\mu_{z}/\sqrt{1-\mu_{z}^{2}} & \mu_{x}/\sqrt{1-\mu_{z}^{2}} & \mu_{y}\\ -\sqrt{1-\mu_{z}^{2}} & 0 & \mu_{z} \end{array}\right]\left[\begin{array}{c} sin\theta_{s}cos\psi_{s}\\ sin\theta_{s}sin\psi_{s}\\ cos\psi_{s} \end{array}\right]$$

If $\left|\mu_{z}\right|\approx 1$, then Equation (11) should be replaced by:

(12) $$\left[\begin{array}{c} \mu_{x}^{\prime }\\ \mu_{y}^{\prime }\\ \mu_{z}^{\prime } \end{array}\right]=\mathrm{sign}\left(\mu_{z}\right)\left[\begin{array}{c} sin\theta_{s}cos\psi_{s}\\ sin\theta_{s}sin\psi_{s}\\ cos\psi_{s} \end{array}\right]$$

### 2.3. Photons Termination

**Figure 1:**
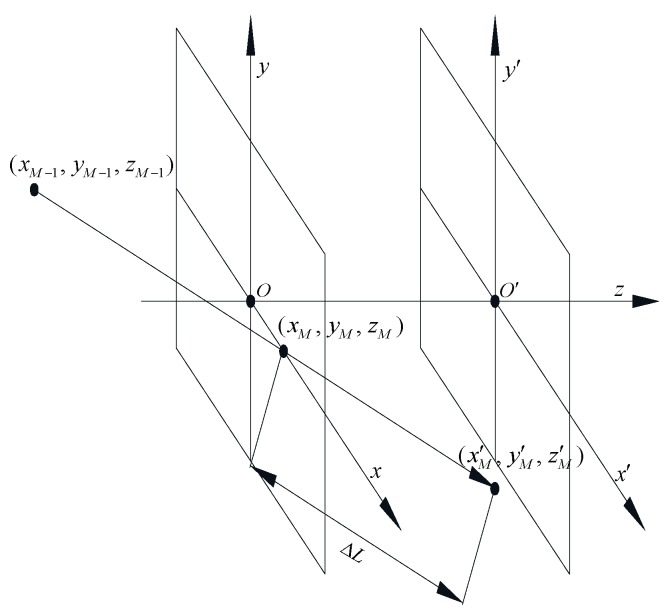
Schematic of arriving coordinates of photons.

Define $Z_{0}$ as the transmission distance, the photon is considered to have arrived at the MC computing plane $\left(x^{\prime }O^{\prime }y^{\prime }\right)$ if Inequality (13) is satisfied, and the tracing of the photon’s propagation is terminated. However, as illustrated in Figure 1, the vector sum of the projections of the propagation distance of photon on the beam axis for each scattering event $L_{z}$ is not always exactly equal to $Z_{0}$. So, when $L_{z}>Z_{0}$, the arriving coordinate deviations of photons caused by ΔL should be modified by Equations (14) and (15), where $\left(x_{M},y_{M},z_{M}\right)$ denotes the arriving coordinates of photons on the receiving plane (xOy), $\left(x_{M}^{\prime },y_{M}^{\prime },z_{M}^{\prime }\right)$ is the MC computing coordinates which on the $x^{\prime }O^{\prime }y^{\prime }$-plane.

(13) $$L_{z}=z_{0}+\sum_{i=1}^{M}\mu_{z_{i}}^{\prime }d_{i}\geq Z_{0}$$

(14) $$\Delta L=\left(L_{z}-Z_{0}\right)/\mu_{z_{M}}^{\prime }$$

(15) $$\left\{\begin{array}{c} x_{M}=x_{0}+\sum_{i=1}^{M}\mu_{x_{i}}^{\prime }d_{i}-\Delta L\mu_{x_{M}}^{\prime }\\ y_{M}=y_{0}+\sum_{i=1}^{M}\mu_{y_{i}}^{\prime }d_{i}-\Delta L\mu_{y_{M}}^{\prime }\\ z_{M}=z_{0}+Z_{0} \end{array}\right.$$

Here, *M* is the total scattering order, $\left(x_{0},y_{0},z_{0}\right)$ is the coordinates of photons emission, $\left(\mu_{x_{i}}^{\prime },\mu_{y_{i}}^{\prime },\mu_{z_{i}}^{\prime }\right)$ is the unit direction vector for the $i^{th}$ scattering event, $d_{i}$ is the propagation distance of the $i^{th}$ scattering event, and $\left(\mu_{x_{M}}^{\prime },\mu_{y_{M}}^{\prime },\mu_{z_{M}}^{\prime }\right)$ is the unit direction vector of the photons’ arrival at the receiving plane.

### 2.4. Photons Reception

The receiving plane is assumed to be a square with its geometric center at the xOy-plane as the origin of the xyz coordinates system, and the laser beam axis as the *z* axis. The half aperture of the receiving plane is $R_{PD}$ and the receiver’s field of view is $\Psi_{R}$. Then, the photons are considered received if Equation (16) is satisfied and the energy weight is greater than the survival threshold.

(16) $$\left\{\begin{array}{c} \left|x_{M}\right|\leq R_{PD}\\ \left|y_{M}\right|\leq R_{PD}\\ L_{z}\geq Z_{0}\\ arccos\left(\mu_{z_{M}}^{\prime }\right)\leq \Psi_{R}/2 \end{array}\right.$$

## 3. The 3 dB Optical Intensity Spot Radius

**Figure 2:**
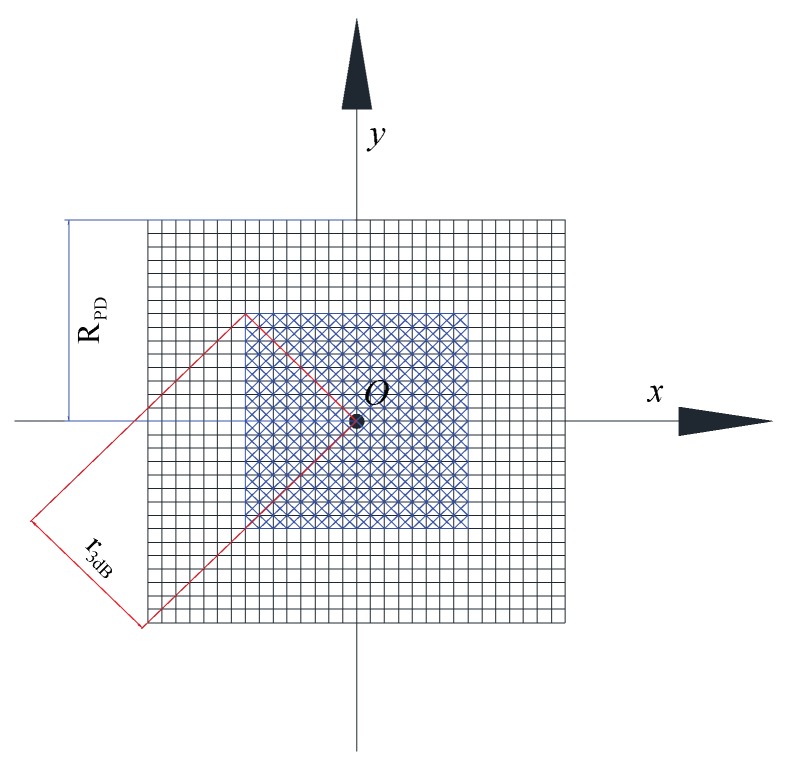
Schematic of optical intensity distribution and 3 dB OISR.

The 2D optical intensity distribution of the laser beam on the receiving plane can be divided into N×N components as shown in Figure 2, i.e., the receiving plane with finite dimensions is divided into N×N infinitesimal square areas, where *N* is a positive integer. The N×N matrix is used to store the intensity information of the laser beam and each element stores the intensity information on each infinitesimal square area. Mark this matrix as Intensity(N,N), and apply Intensity(η,ζ) to store the intensity distributed on the NO.(η,ζ) infinitesimal square area. So, the analysis for optical intensity distribution on the receiving plane can be equivalent to conducting algebraic operations on Intensity(N,N), and the total intensity is presented by Equation (17):

(17) $$R_{Inty}=\sum_{\eta =1}^{N}\sum_{\zeta =1}^{N}Intensity\left(\eta ,\zeta \right)$$

(18) $$10\mathrm{lg}\left(\frac{\sum_{l=N/2-N_{3dB}+1}^{N/2+N_{3dB}}\sum_{p=N/2-N_{3dB}+1}^{N/2+N_{3dB}}Intensity\left(l,p\right)}{R_{Inty}}\right)\geq -3$$

Solving the minimum integer of $N_{3dB}\left(N_{3dB}<N/2\right)$, which makes the Inequality (18) hold, the 3 dB OISR $\left(r_{3dB}\right)$ which is the key parameter of this paper is given by:

(19) $$r_{3dB}=\sqrt{2}N_{3dB}R_{PD}/N$$

## 4. Numerical Results and Analysis

Based on the aforementioned MC simulation method and the computational principle, this paper analyzes the characteristics of the 3 dB OISR of the laser beam under various seawater channels. A laser source with a beam waist radius of $5\times 10^{-3}$ m, and divergence angle (T−Phai) of 1, 5 and 10 mrad is considered. It is assumed that the photons transmission velocity in seawater is $0.75\times 2.9979\times 10^{8}$ m/s, the asymmetry parameter g=0.924 and simulation photons quantity is $5\times 10^{7}$. Table 2 shows the other important channel parameters used in the simulation.

To study the effects of detection aperture on $r_{3dB}$, eight-channel types are investigated: 44 and 52 m pure seawater (Pur-44, Pur-52), 34 and 42 m clean seawater (Cle-34, Cle-42), 24 and 32 m coastal seawater (Coa-24, Coa-32) and 6 and 8 m harbor seawater (Har-6, Har-8). To further reveal the impacts of transmission distances on $r_{3dB}$, the transmission distances of $Z_{0}\leq 160$ m, $Z_{0}\leq 70$ m, $Z_{0}\leq 35$ m and $Z_{0}\leq 10$ m, respectively, for pure, clean, coastal and harbor seawater channel are investigated. The transmission distance is varied for different seawater channels to account for the difference in attenuation per unit distance (attenuation coefficient) (see Table 2).

To describe the trend of $r_{3dB}$ as a function of the half-aperture of the receiving plane and transmission distance, based on the MC simulation data of $r_{3dB}$, the K−term Gaussian function (K=3,4,5,6,7) shown by Equation (20) is utilized for curve fitting.

(20) $$r_{3dB-Fitting}\left(X\right)=\sum_{n=1}^{K}a_{n}exp\left[-{\left(\frac{X-b_{n}}{c_{n}}\right)}^{2}\right]$$

where *X* denotes $R_{PD}$ or $Z_{0}$ which depends on the MC simulation data types, the value of *K* is determined by the fitting accuracy, $a_{n}$, $b_{n}$ and $c_{n}$ are the fitting coefficients of the K−term Gaussian functions.

### 4.1. Pure Seawater Channel

**Figure 3:**
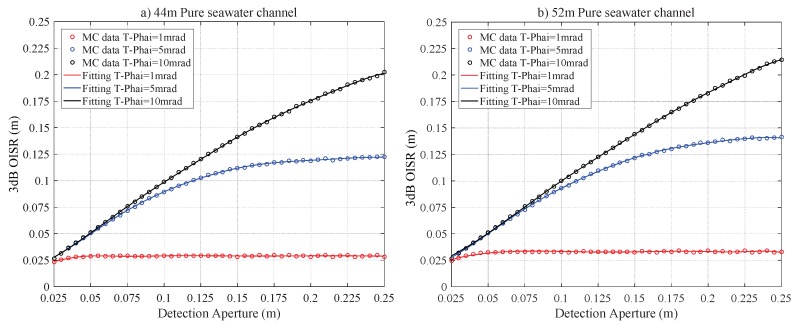
The $r_{3dB}$ versus $R_{PD}$ for pure seawater laser channel for a link distance of (**a**) 44 m and (**b**) 52 m.

**Figure 4:**
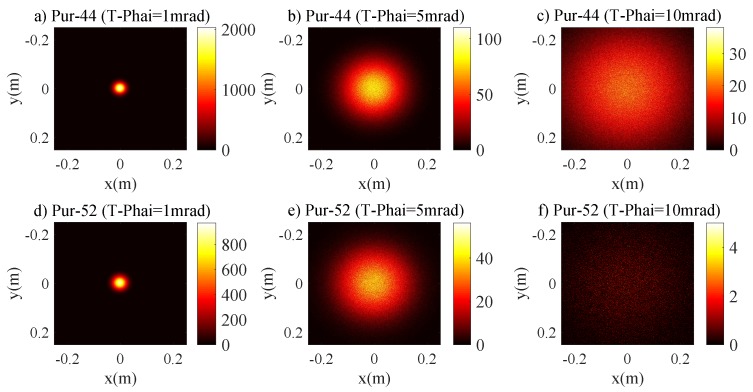
2D intensity distribution of laser beam for pure seawater channel (**a**) Pur-44 with T-Phai = 1 mrad, (**b**) Pur-44 with T-Phai = 5 mrad, (**c**) Pur-44 with T-Phai = 10 mrad, (**d**) Pur-52 with T-Phai = 1 mrad, (**e**) Pur-52 with T-Phai = 5 mrad and (**f**) Pur-52 with T-Phai = 10 mrad.

The relations between $r_{3dB}$ of the laser beam and $R_{PD}$ are given by Equations (17), (19) and Inequality (18). Figure 3 presents the relations between $r_{3dB}$ and $R_{PD}$ for Pur-44 and Pur-52 channels. There is an approximately linear relationship between $r_{3dB}$ and $R_{PD}$ initially. Then the $r_{3dB}$ saturates and hence it does not increase with the $R_{PD}$. The saturation effect is clearer for a small T−Phai = 1 and 5 mrad. This can be attributed to the fact that the role of the scattering on the laser beam intensity distribution is negligible and the beam spot size is dominantly determined by the natural divergence of the laser source. For the Pur-44 channel and the laser source with T−Phai = 1 mrad, the beam area can be completely collected at the receiving plane, and $r_{3dB}\approx$ 0.0290 m is the saturation value if $R_{PD}\geq 0.065$ m. Figure 4 shows the 2D intensity distribution of the laser beam for T−Phai = 1, 5 and 10 mrad under Pur-44 and Pur-52 channels. The Gaussian characteristics expected for the laser beam are well maintained. This clearly indicates that the scattering has a negligible effect on the laser beam intensity distribution. The $r_{3dB}$ values of the Pur-52 channel are slightly greater than the Pur-44 channel. This is expected as the longer transmission distance leads to greater divergence and hence a larger beam area.

**Figure 5:**
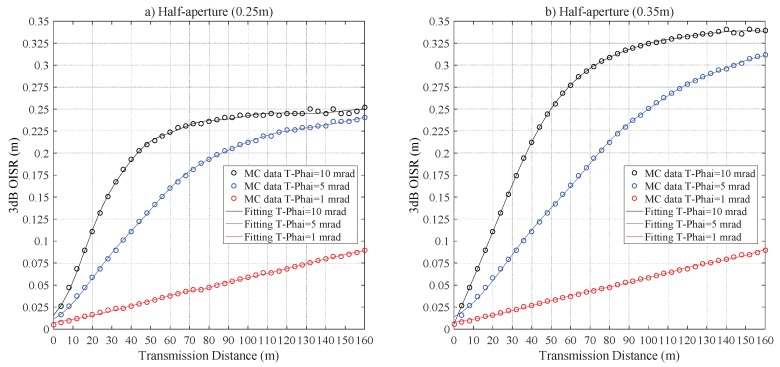
The $r_{3dB}$ versus $Z_{0}$ for pure seawater laser channel for a half-aperture of (**a**) $R_{PD}$ = 0.25m and (**b**) $R_{PD}$ = 0.35 m.

Figure 5 illustrates the impacts of $Z_{0}$ on $r_{3dB}$. There is an approximately linear relationship between $r_{3dB}$ and $Z_{0}$ for a very short transmission distance. Then the $r_{3dB}$ diverges from a linear relationship, and ultimately, the $r_{3dB}$ saturates and hence it does not increase with $Z_{0}$. The saturation effect is clearer for T-Phai = 10 mrad, this is due to the fact that the larger divergence angle will lead to higher geometric loss, which means the receiver can only collect the center portion of the scattered and direct arrival photons. For the configurations of $R_{PD}$ = 0.25 m and 0.35 m with T−Phai = 10 mrad, $r_{3dB}\approx 0.0242$ m and 0.3410 m are the saturation values for $Z_{0}\geq$ 112 m and 140 m, respectively. For the configurations of $R_{PD}$ = 0.25 m and 0.35 m and T−Phai = 1 mrad, the two curves almost overlapped; this is due to the fact that the laser beam radius is less than 0.25 m and this makes the whole laser beam spot able to be covered by the receiver if $R_{PD}\geq$ 0.25 m and $Z_{0}\leq$ 160 m.

### 4.2. Clean Seawater Channel

**Figure 6:**
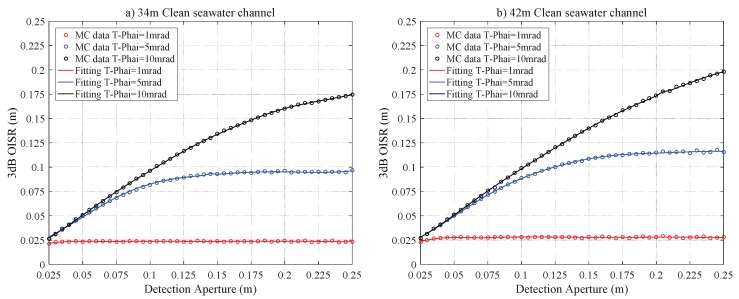
The $r_{3dB}$ versus $R_{PD}$ for the clean seawater laser channel for a link distance of (**a**) 34 m and (**b**) 42 m.

**Figure 7:**
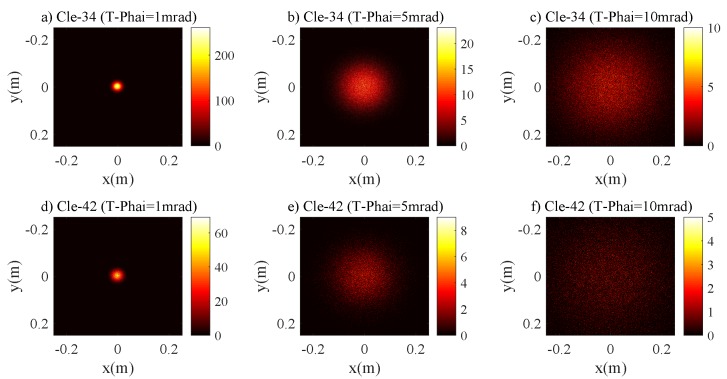
The 2D intensity distribution of the laser beam for the clean seawater channel (**a**) Cle-34 with T-Phai = 1 mrad, (**b**) Cle-34 with T-Phai = 5 mrad, (**c**) Cle-34 with T-Phai = 10 mrad, (**d**) Cle-42 with T-Phai = 1 mrad, (**e**) Cle-42 with T-Phai = 5 mrad and (**f**) Cle-42 with T-Phai = 10 mrad.

**Figure 8:**
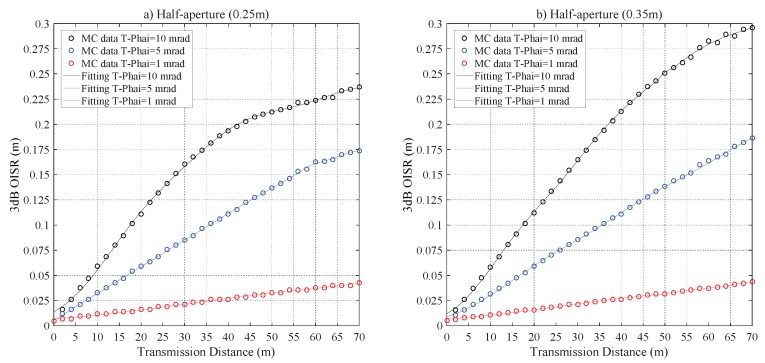
The $r_{3dB}$ versus $Z_{0}$ for the clean seawater laser channel for a half-aperture of (**a**) $R_{PD}$ = 0.25 m and (**b**) $R_{PD}$ = 0.35 m.

Figure 6 characterizes the relations between $r_{3dB}$ and $R_{PD}$ for the Cle-34 and Cle-42 channels. The curves trend is similar to the case of the pure seawater channel. For the Cle-34 and Cle-42 channels with T−Phai = 1 mrad, $r_{3dB}\approx 0.0237$ m and 0.0280 m, respectively, are the saturation value if $R_{PD}\geq 0.075$ m. For the laser source with T−Phai = 5 mrad, these values are $r_{3dB}\approx 0.0960$ m and 0.1150 m with $R_{PD}\geq 0.175$ m and $R_{PD}\geq 0.200$ m, respectively, for the Cle-34 and Cle-42 channels. The results show that the influences of scattering on the laser beam intensity distribution are still negligible and the receiving plane can collect the whole beam spot. Figure 7 also demonstrates that 2D intensity distribution of the laser beam is Gaussian, and clearly illustrates that the scattering effects on the laser beam are insignificant. Figure 8 characterizes the the impacts of $Z_{0}$ on $r_{3dB}$; the curves trend is similar to Figure 5. As in the case of the pure seawater channel, for the configurations of $R_{PD}$ = 0.25 m and 0.35 m and T−Phai = 1 mrad, the two curves of $r_{3dB}$ almost overlapped. This is due to the fact that the geometric loss still plays a very important role in $r_{3dB}$.

### 4.3. Coastal Seawater Channel

**Figure 9:**
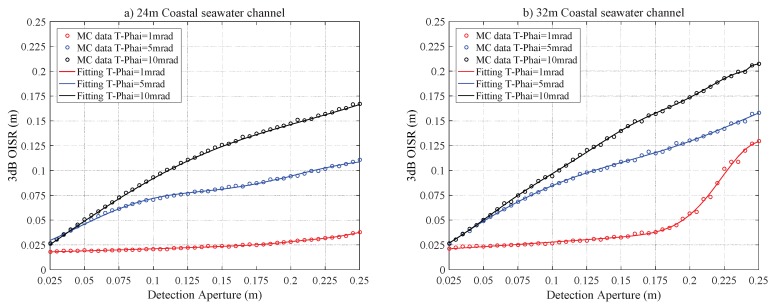
The $r_{3dB}$ versus $R_{PD}$ for the coastal seawater laser channel for a link distance of (**a**) 24 m and (**b**) 32 m.

**Figure 10:**
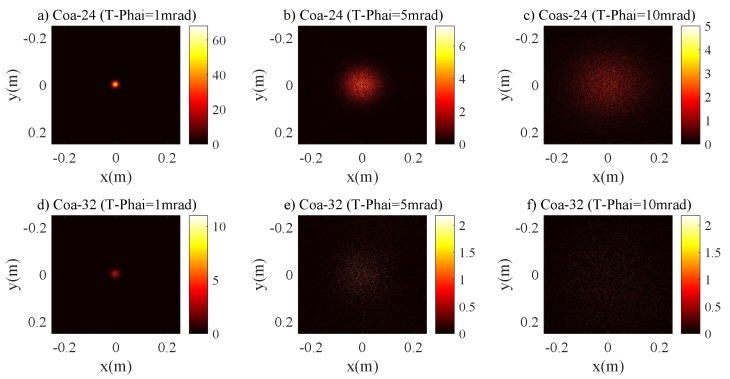
The 2D intensity distribution of the laser beam for coastal seawater channel (**a**) Coa-24 with T-Phai = 1 mrad, (**b**) Coa-24 with T-Phai = 5 mrad, (**c**) Coa-24 with T-Phai = 10 mrad, (**d**) Coa-32 with T-Phai = 1 mrad, (**e**) Coa-32 with T-Phai = 5 mrad and (**f**) Coa-32 with T-Phai = 10 mrad.

Figure 9 depicts the relations between $r_{3dB}$ and $R_{PD}$ for the Coa-24 and Coa-32 channels. The curves trend shows a significant deviation from the previous two cases of pure and clean seawater channels. For example, for the case of the Coa-32 channel with T−Phai = 1 mrad, $r_{3dB}$ diverges from a linear relationship when $R_{PD}>0.165$ m. This is because the laser beam is scattered sparsely around the spot center, which leads to the evident increase in the $r_{3dB}$. For the case of T−Phai = 5 and 10 mrad, the curves show approximately a linearity trend initially and then saturate slowly. This is due to the consequence of increased density of scattering particles in the coastal seawater channel, resulting in a higher scattering probability of photons and causing the propagation trajectory of photons to deviate from the original direction. This leads to the non-Gaussian intensity distribution on the receiving plane. Figure 10 shows the 2D intensity distribution of the laser beam with a divergence angle of T−Phai = 1, 5 and 10 mrad under Coa-24 and Coa-32 channels. The figure clearly shows that Gaussian characteristics expected for the laser beam are destroyed seriously. Hence the relationship between $r_{3dB}$ and $R_{PD}$ is no longer similar to the cases of pure and clean seawater channels.

**Figure 11:**
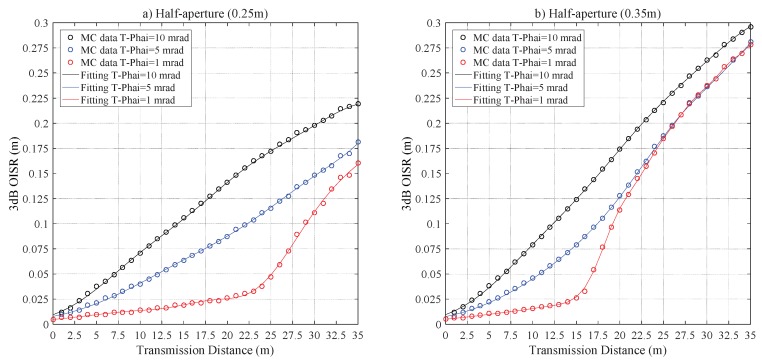
The $r_{3dB}$ versus $Z_{0}$ for coastal seawater laser channel for a half-aperture of (**a**) $R_{PD}$ = 0.25 m and (**b**) $R_{PD}$ = 0.35 m.

It is clear from Figure 11, that the curves $r_{3dB}$ with $Z_{0}$ diverges significantly from the linear relationship for the laser beam with T−Phai = 1 mrad, and $r_{3dB}\approx$ 0.0259 m with $Z_{0}$ = 21 m, $r_{3dB}\approx$ 0.0647 m with $Z_{0}$ = 14 m are the divergence points, respectively, for $R_{PD}$ = 0.25 m and 0.35 m. The curves almost overlap for the laser beam with T−Phai = 1 and 5 mrad if $Z_{0}\geq$ 25 m with $R_{PD}$ = 0.35 m. This is caused by the effects of increased scattering events, which weakens the role of geometric loss in $r_{3dB}$ with the increase of $Z_{0}$.

### 4.4. Harbor Seawater Channel

**Figure 12:**
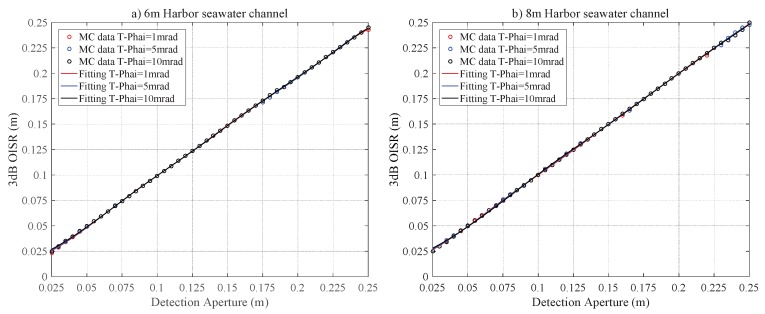
The $r_{3dB}$ versus $R_{PD}$ for harbor seawater laser channel for a link distance of (**a**) 6 m and (**b**) 8 m.

**Figure 13:**
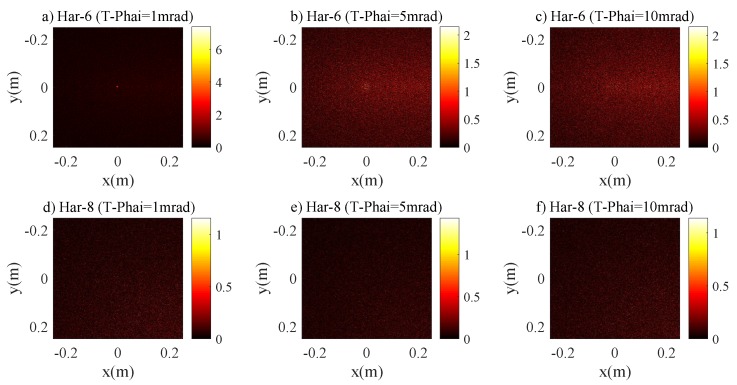
The 2D intensity distribution of a laser beam for harbor seawater channel (**a**) Har-6 with T-Phai = 1 mrad, (**b**) Har-6 with T-Phai = 5 mrad, (**c**) Har-6 with T-Phai = 10 mrad, (**d**) Har-8 with T-Phai = 1 mrad, (**e**) Har-8 with T-Phai = 5 mrad and (**f**) Har-8 with T-Phai = 10 mrad.

Figure 12 describes the relations between $r_{3dB}$ and $R_{PD}$ for the Har-6 and Har-8 channels. Unlike the previous cases, the $r_{3dB}$ does not depend on the divergence angle of the laser sources indicating the dominance of scattering in the 3 dB optical intensity spot. The harbor seawater contains a significantly higher concentration of scattering particles than the coastal seawater channel. Consequently, almost 100% of photons are scattered and hence the optical intensity at the receiving plane is randomly distributed. Figure 13 shows the 2D intensity distribution of the laser beam with the divergence angle T−Phai = 1, 5 and 10 mrad under Harbor channel. This shows that the 2D intensity distribution of the laser beam does not depend on the divergence angle and the intensity distribution is distributed randomly instead of Gaussian.

**Figure 14:**
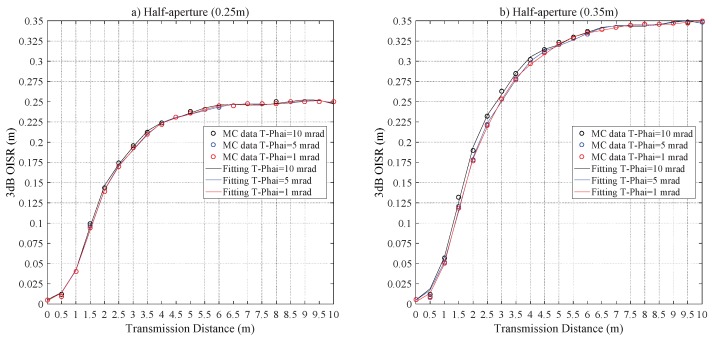
The $r_{3dB}$ versus $Z_{0}$ for the harbor seawater laser channel for a half-aperture of (**a**) $R_{PD}$ = 0.25 m and (**b**) $R_{PD}$ = 0.35 m.

**Figure 15:**
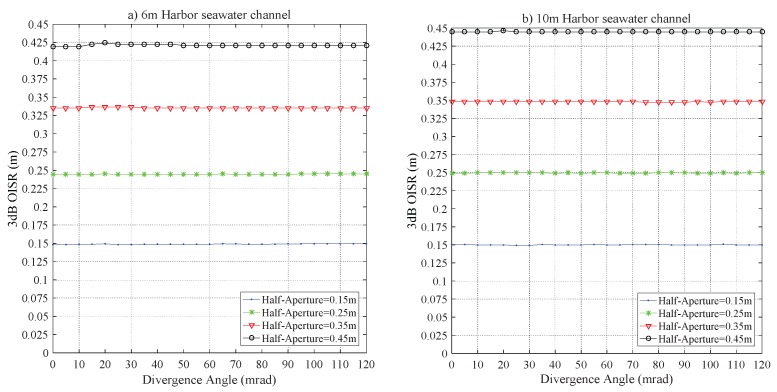
The $r_{3dB}$ versus T-Phai for the harbor seawater laser channel for a link distance of (**a**) 6 m and (**b**) 10 m.

Figure 14 demonstrates the impact of $Z_{0}$ on $r_{3dB}$. For the configurations of $R_{PD}$ = 0.25 m and 0.35 m, the difference of $r_{3dB}$ is less than 0.0013 m and 0.0047 m, respectively; the saturation values of $r_{3dB}$, respectively, are 0.2498 m and 0.3485 m with $Z_{0}\geq$ 6 m. Additionally, to further address the 2D distribution of the laser beam in the harbor seawater channel. Figure 15 shows the relations of $r_{3dB}$ and T−Phai, respectively, for $Z_{0}$ = 6 m and 10 m, which distinctly demonstrates that the divergence angle plays a negligible role in $r_{3dB}$.

### 4.5. Verification for the 3 dB Optical Intensity

The 3 dB OISR proposed in this paper is computed based on Equations (17)–(19). Hence, to verify the validity of the $r_{3dB}$, the attenuation loss (in dB) of total optical intensity that is covered by the $r_{3dB}$ versus the total received optical intensity on the receiving plane is calculated. All the calculated attenuation loss values satisfy Inequality (18), and the difference is less than −2.5 dB. This shows that the calculated $r_{3dB}$ in this paper can cover the dominating optical intensity of the laser beam.

## 5. Conclusions

In this paper, the underwater optical channel is characterized based on optical intensity distribution and 3 dB optical intensity spot radius for the first time. The 3 dB optical intensity spot radius is an important parameter for underwater laser communication system design as most of the optical power is concentrated within this radius, and the aperture of the real optical receiver tends to be significantly smaller than the radius of the whole beam spot at the receiving plane. Hence, the 3 dB optical intensity spot radius is calculated for various underwater optical channels. In the investigation, the Henyey–Greenstein function is used to calculate the scattering angles of photons, and the influences of the underwater optical channel and optical system parameters on the 3 dB optical intensity spot radius are studied based on the Monte Carlo simulation method. The study found that there is an approximately linear relationship between $r_{3dB}$ and $R_{PD}$ initially for a channel with less density of scattering particles (such as clean water). Then, the $r_{3dB}$ saturates and does not increase with the $R_{PD}$. Furthermore, there is approximately a linear relationship between $r_{3dB}$ and $Z_{0}$ initially, then the $r_{3dB}$ diverges from a linear relationship, and ultimately saturates and does not increase with the $Z_{0}$. Additionally, the verification shows that the calculated $r_{3dB}$ in this paper can cover the dominating optical intensity of the laser beam. For a highly scattering channel (such as the harbor channel), the optical intensity distribution is no longer a Gaussian and the effects of the divergence angle on $r_{3dB}$ are negligible. Hence, for a highly scattering channel, the design of receiver aperture and optimal spacing of the diversity scheme for the underwater laser communication systems cannot be predicted based the divergence angle and geometric loss.

## Figures and Tables

**Table 1:** Survey of recent underwater optical channel modeling.

Ref. No.	Methods	Contribution Highlights
[4]	VRT theory	Path losses. Received waveform degradation. Link bit error rate.
[5]	BSF	Optical power distribution on the receiving plane.
[6,7,8]	Experiments	Modulation depth, degree of polarization of modulated light.
[9]	MC	CIR. Channel capacity.
[10]	MC	Path losses. CIR. Bit error rate. Received photons distribution.
[11]	Experiments	Effects of misalignment, scattering agents on temporal response.
[12]	MC	Path losses for various channel configurations.
[13]	MC	Wavelength-dependent path losses based on the bio-optical model of seawater given by [14].
[15]	RTE	Path losses modeled by direct RTE solver.
[16]	Closed expression	CIR modeled by double gamma functions.
[17]	Closed expression	MIMO CIR modeled by weight gamma function polynomial.
[18]	Stochastic model	Spatial and temporal probability characteristics of photons.
[19]	Closed expression	Path losses modeled by weighted function of two exponentials.
[20]	MC	CIR and normalized received optical power.
[21]	MC	Different effects of two scattering angle computational principle on CIR.
[22]	Experiments	Statistical distribution of optical intensity fluctuations caused by temperature-induced oceanic turbulence.
[23]	MC	Probability density function of oceanic turbulence channel. Turbulence-induced scintillation index and path losses.
[24]	MC	Empirical model of transmission distance-dependent path losses.
[25]	MC	Channel estimation and evaluation under geometric losses.
[26]	MC	Scattering regimes of photons.
[27]	MC	Optical receiving power, CIR based on a newly developed scattering phase function which better fit for real seawater.
[28]	Experiments	Statistical model of intensity fluctuations caused by random temperature and salinity variations and air bubbles. Channel coherence time.
[29]	Closed expression	New CIR model that is superior to the weighted double gamma functions.
[30]	Ray tracing	CIR and path losses for blocking and shadowing channel.
[31]	Modified BL law	Path losses.
[32]	Experiments	Air bubble and temperature gradient-induced channel irradiance fluctuations presented by mixture exponential-generalized gamma distribution.
[33]	Numerical Model	Influences of group velocity dispersion and time jitter at the pulse width, probability fade and maximum bit rate.
[34]	BSF	Lower mathematical complexity and simplicity.
[35]	RTE	Improved accurate solver for time-dependent RTE.
[36]	Experiments	Beam’s wave-front distortion caused by turbulence. Real-time associated Zernike coefficients. Transmission of polarized light and light with OAM.
[37]	Experiments	Impacts of temperature gradient-induced turbulence, population and size of air bubbles on non-line-of-sight channel.

**Table 2:** Underwater optical channel parameters based on [10].

Items	Channel Parameters			
	Pure	Clean	Coastal	Harbor
$K_{a}\left(\lambda \right)(m^{-1})$	0.053	0.069	0.088	0.295
$K_{s}\left(\lambda \right)(m^{-1})$	0.003	0.080	0.216	1.875
$K_{att}\left(\lambda \right)(m^{-1})$	0.056	0.150	0.305	2.170
